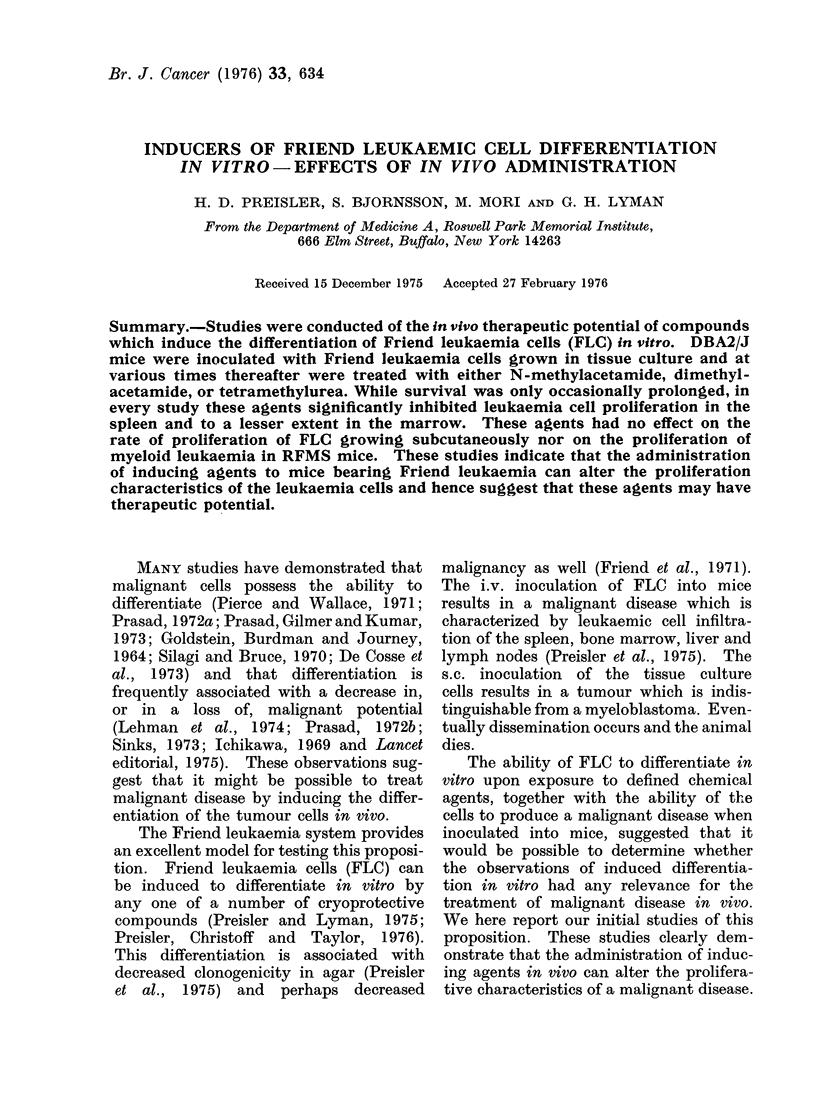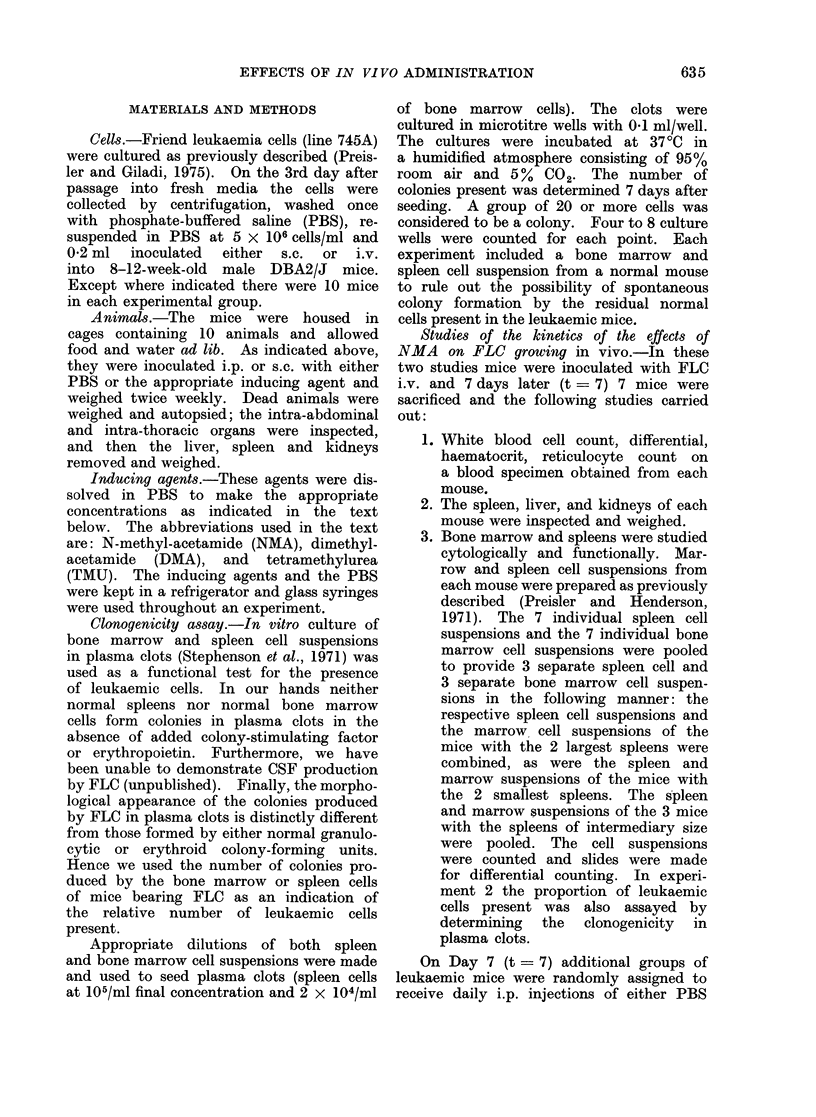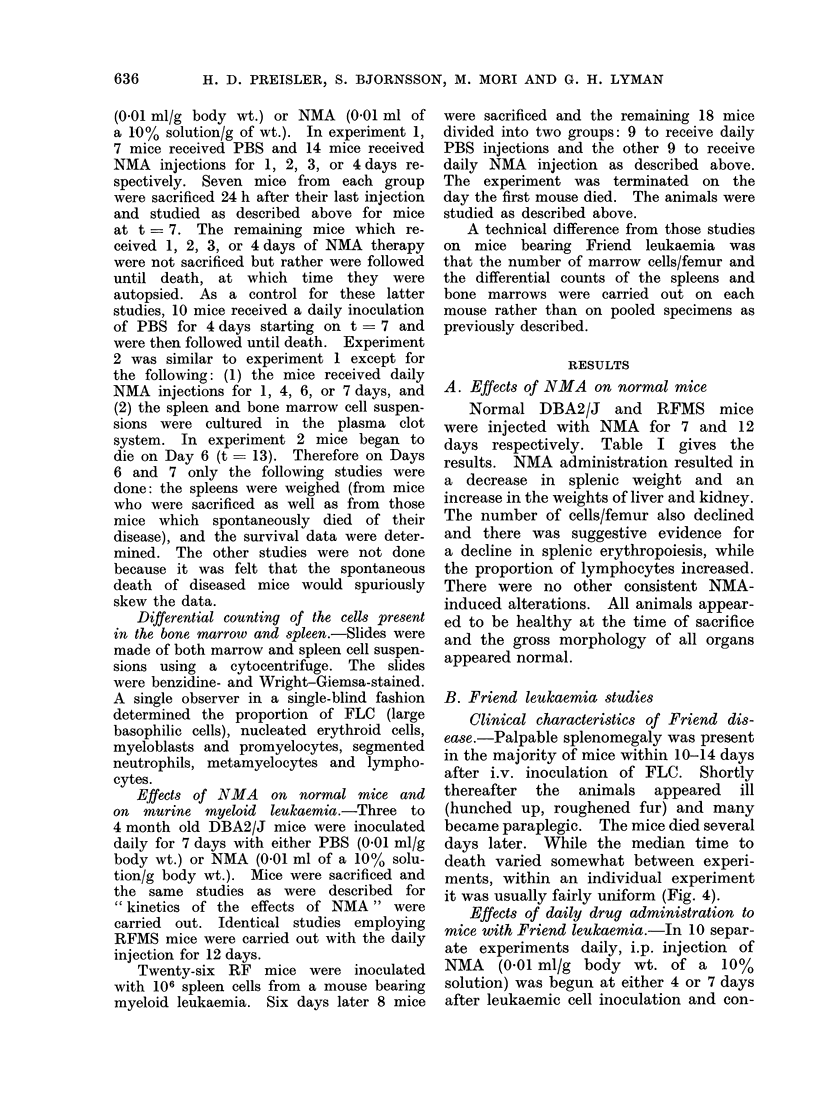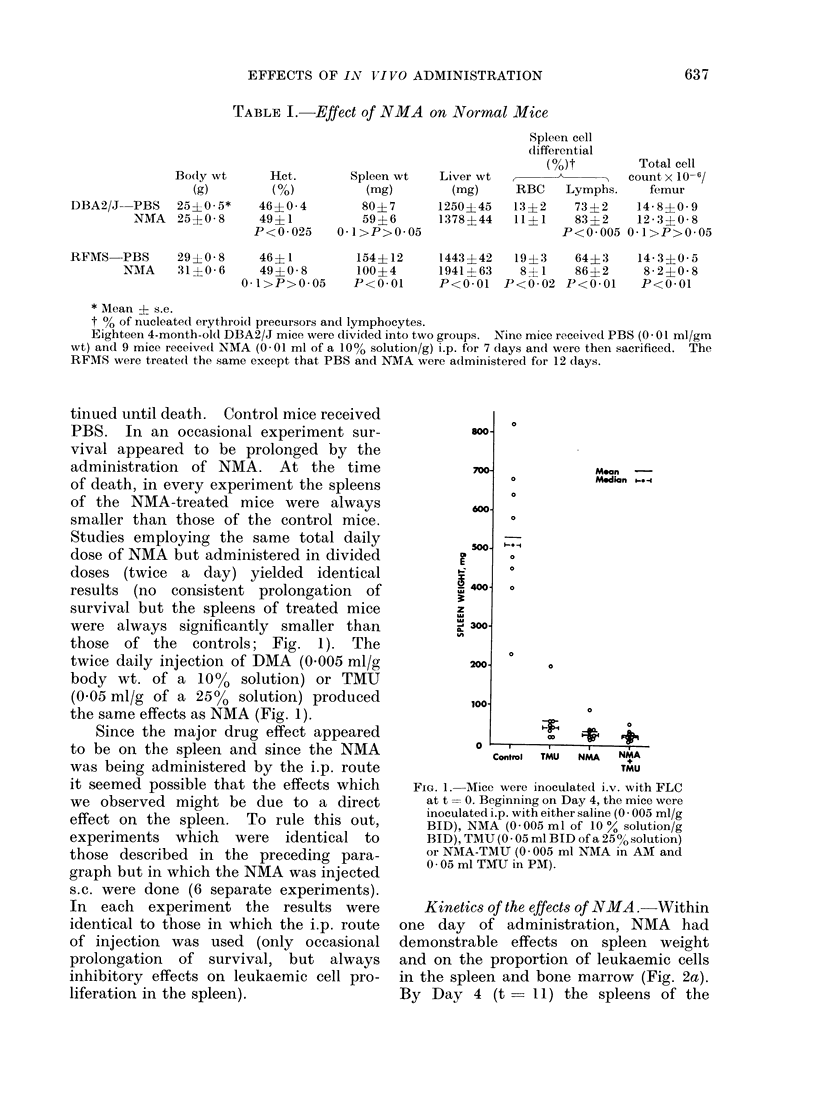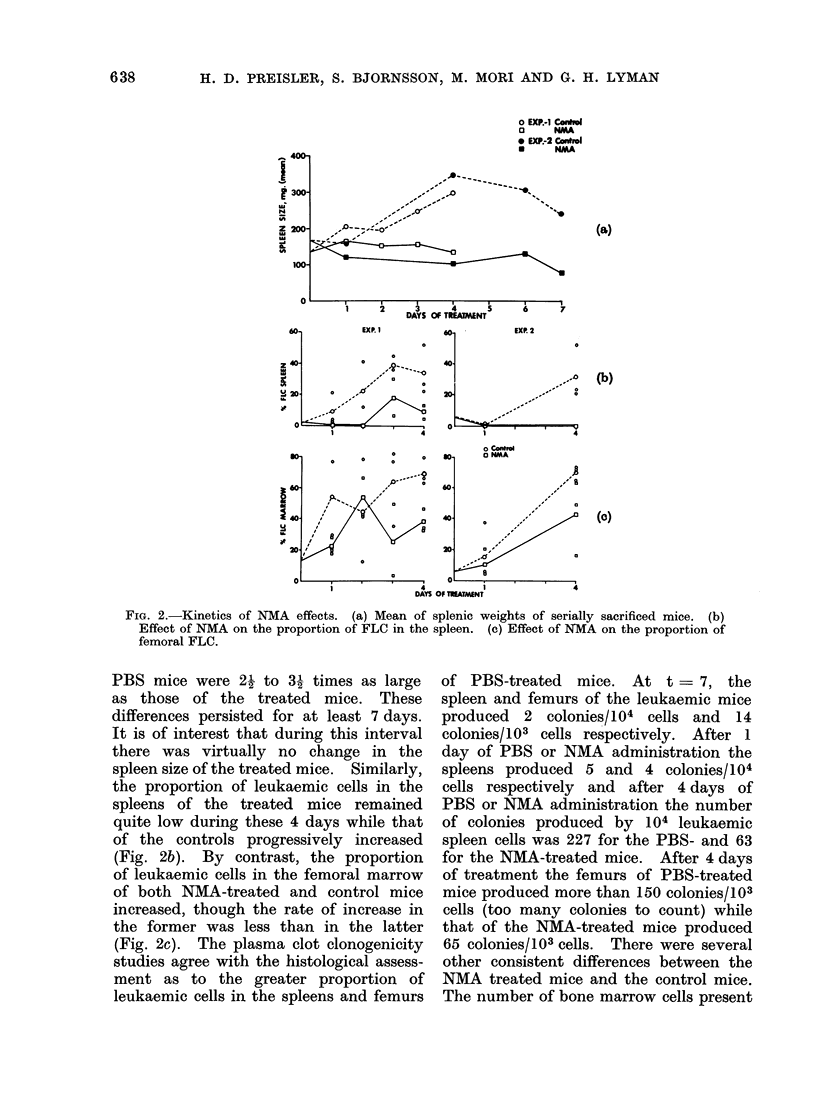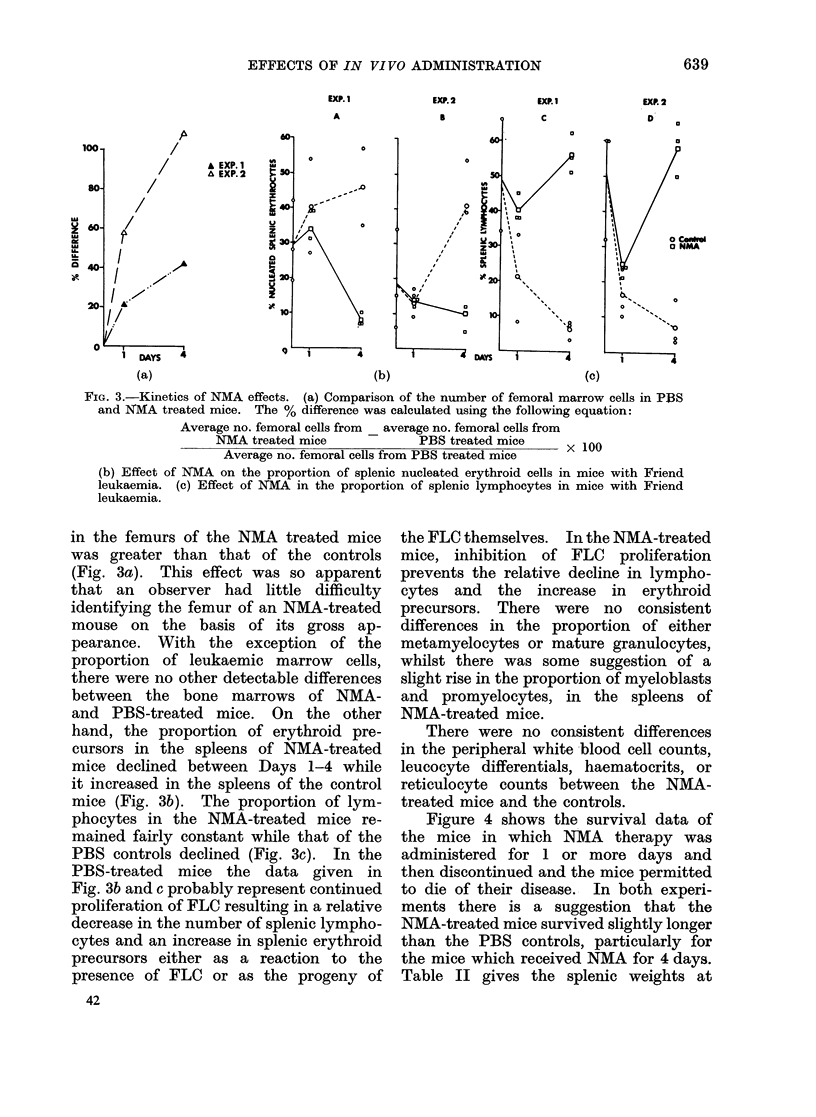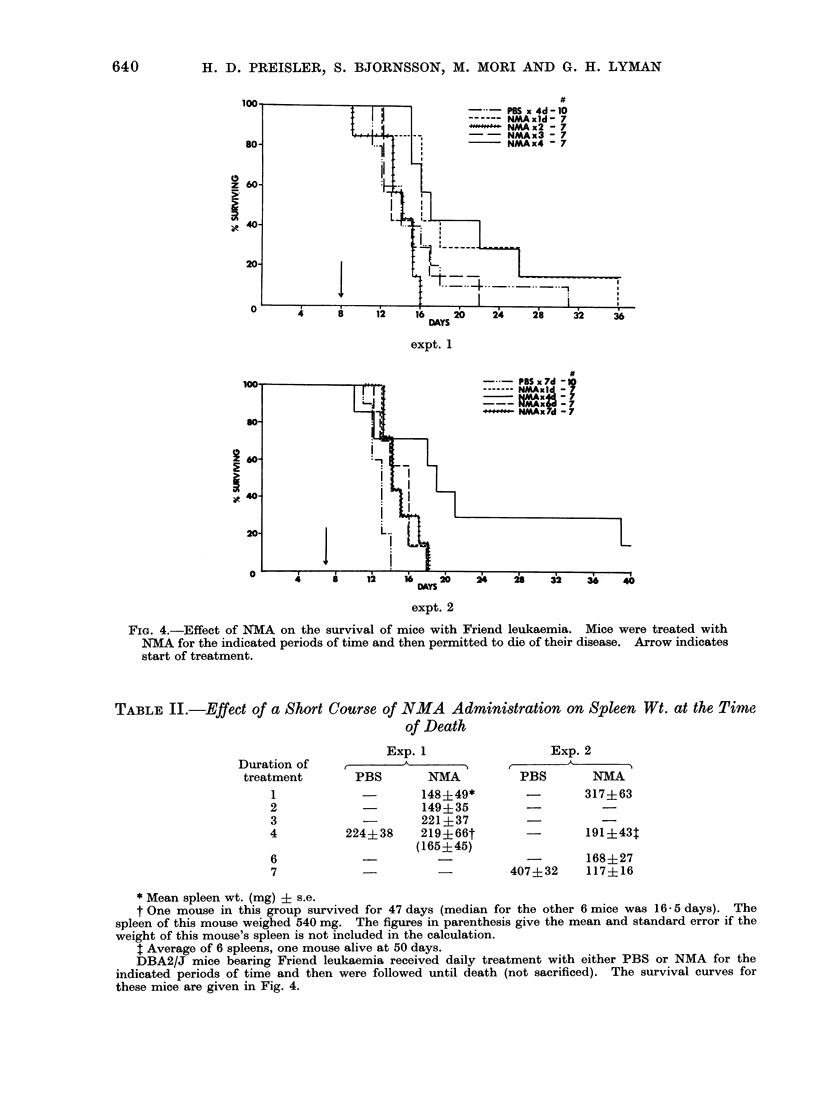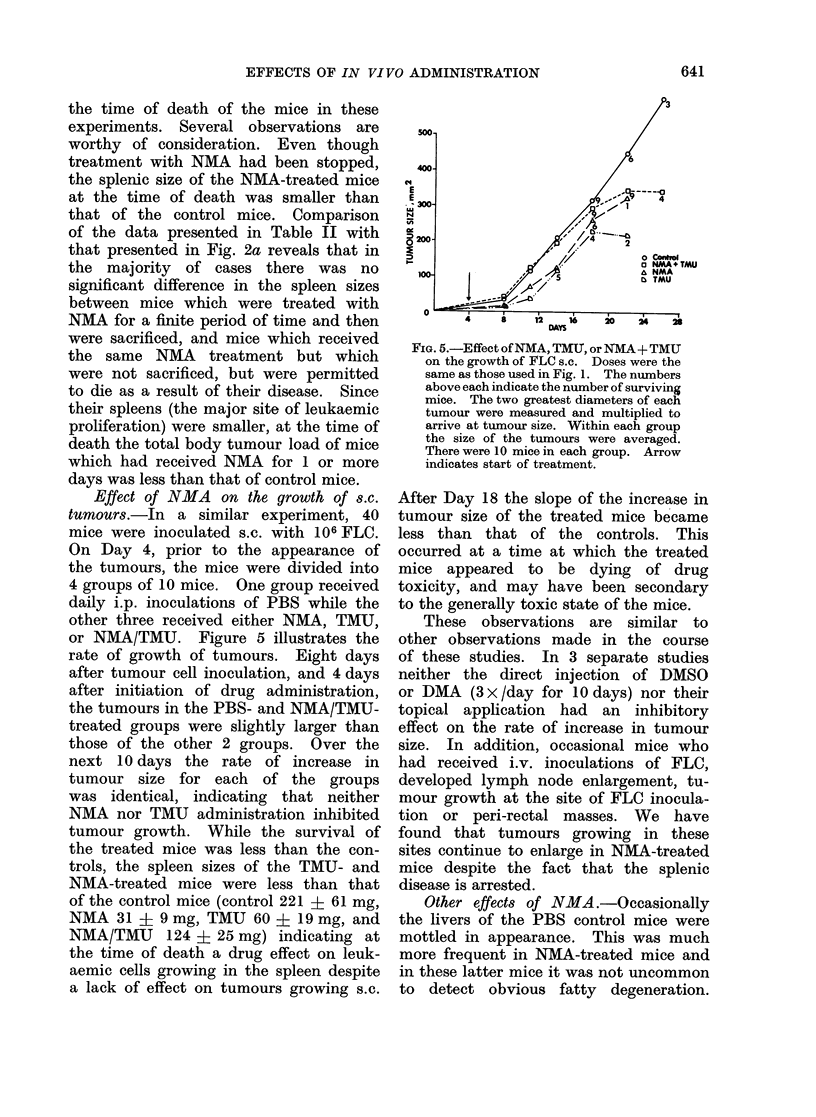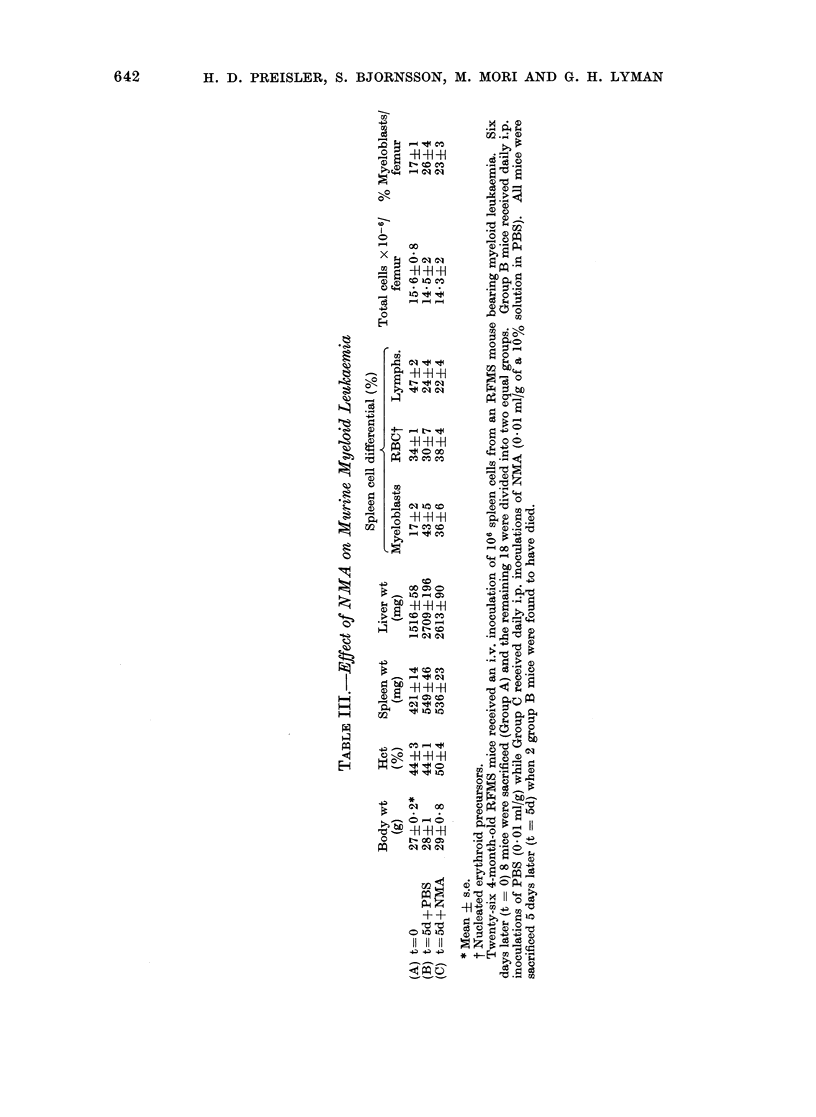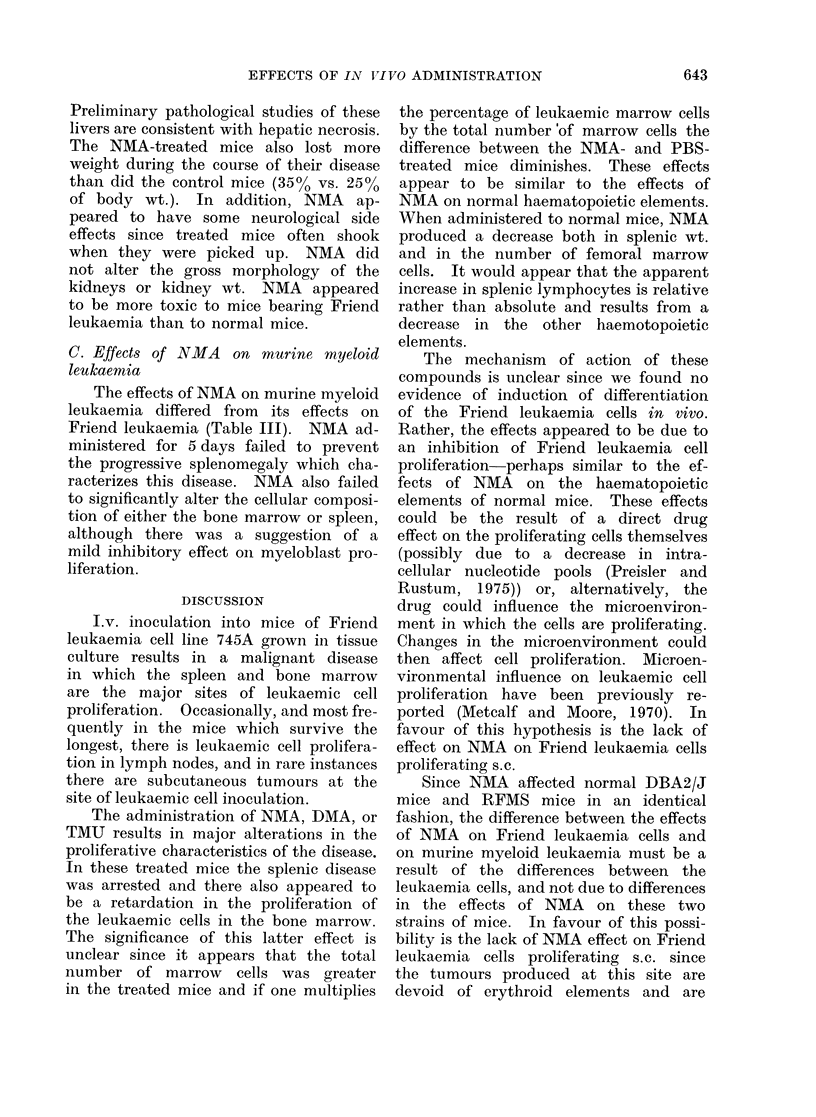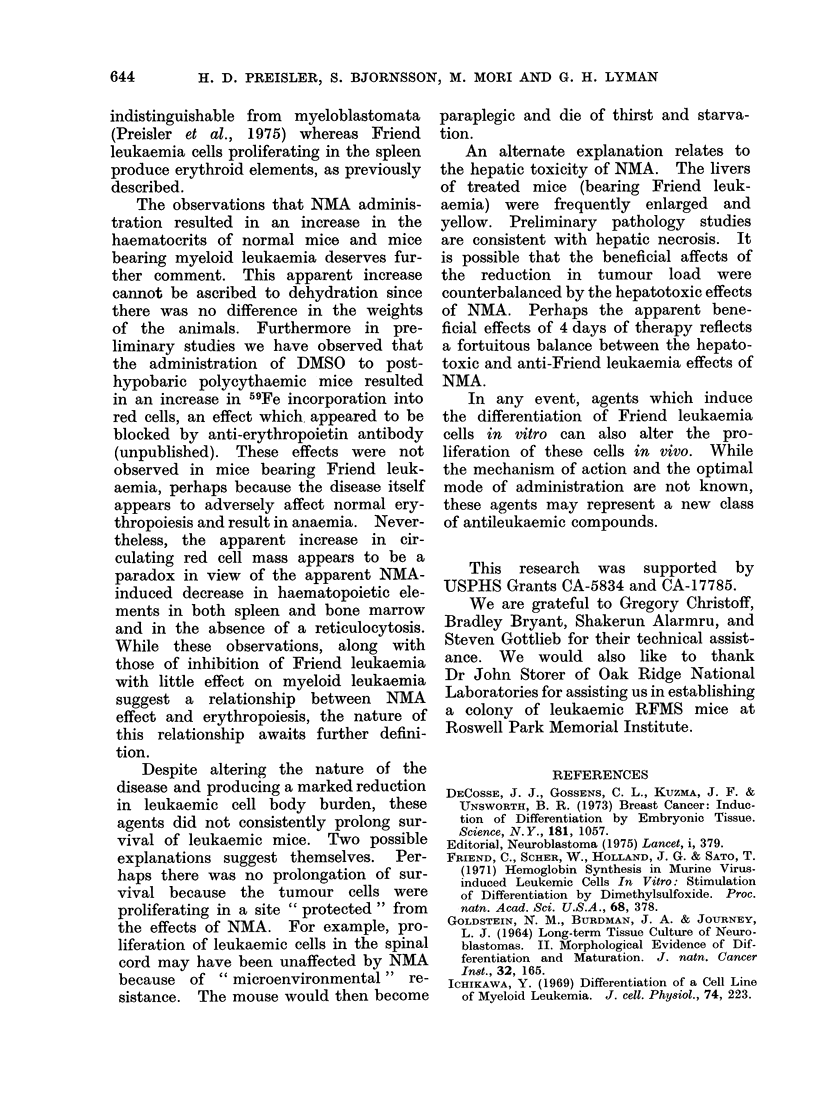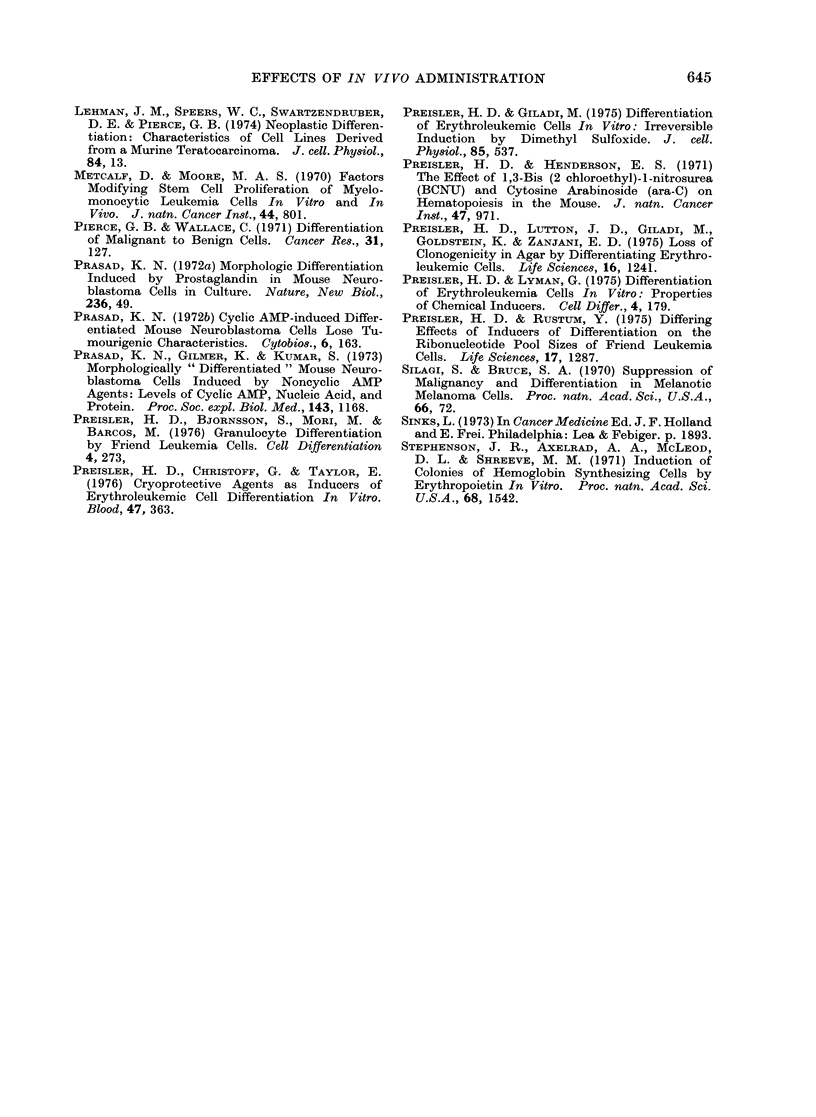# Inducers of Friend leukaemic cell differentiation in vitro--effects of in vivo administration.

**DOI:** 10.1038/bjc.1976.101

**Published:** 1976-06

**Authors:** H. D. Preisler, S. Bjornsson, M. Mori, G. H. Lyman

## Abstract

Studies were conducted of the in vivo therapeutic potential of compounds which induce the differentiation of Friend leukaemia cells (FLC) in vitro. DBA2/J mice were inoculated with Friend leukaemia cells grown in tissue culture and at various times thereafter were treated with either N-methylacetamide, dimethylacetamide, or tetramethylurea. While survival was only occasionally prolonged, in every study these agents significantly inhibited leukaemia cell proliferation in the spleen and to a lesser extent in the marrow. These agents had no effect on the rate of proliferation of FLC growing subcutaneously nor on the proliferation of myeloid leukaemia in RFMS mice. These studies indicate that the administration of inducing agents to mice bearing Friend leukaemia can alter the proliferation characteristics of the leukaemia cells and hence suggest that these agents may have therapeutic potential.


*Br. J. Cancer* (1976) **33**, 634

### INDUCERS OF FRIEND LEUKAEMIC CELL DIFFERENTIATION *IN VITRO*—EFFECTS OF *IN VIVO* ADMINISTRATION

H. D. PREISLER, S. BJORNSSON, M. MORI AND G. H. LYMAN

*From the Department of Medicine A, Roswell Park Memorial Institute,*
666 *Elm Street, Buffalo, New York* 14263

Received 15 December 1975 Accepted 27 February 1976

**Summary.—Studies were conducted of the *in vivo* therapeutic potential of compounds which induce the differentiation of Friend leukaemia cells (FLC) *in vitro*. DBA2/J mice were inoculated with Friend leukaemia cells grown in tissue culture and at various times thereafter were treated with either N-methylacetamide, dimethylacetamide, or tetramethylurea. While survival was only occasionally prolonged, in every study these agents significantly inhibited leukaemia cell proliferation in the spleen and to a lesser extent in the marrow. These agents had no effect on the rate of proliferation of FLC growing subcutaneously nor on the proliferation of myeloid leukaemia in RFMS mice. These studies indicate that the administration of inducing agents to mice bearing Friend leukaemia can alter the proliferation characteristics of the leukaemia cells and hence suggest that these agents may have therapeutic potential.**

MANY studies have demonstrated that malignant cells possess the ability to differentiate (Pierce and Wallace, 1971; Prasad, 1972*a*; Prasad, Gilmer and Kumar, 1973; Goldstein, Burdman and Journey, 1964; Silagi and Bruce, 1970; De Cosse *et al.*, 1973) and that differentiation is frequently associated with a decrease in, or in a loss of, malignant potential (Lehman *et al.*, 1974; Prasad, 1972*b*; Sinks, 1973; Ichikawa, 1969 and *Lancet* editorial, 1975). These observations suggest that it might be possible to treat malignant disease by inducing the differentiation of the tumour cells *in vivo*.

The Friend leukaemia system provides an excellent model for testing this proposition. Friend leukaemia cells (FLC) can be induced to differentiate *in vitro* by any one of a number of cryoprotective compounds (Preisler and Lyman, 1975; Preisler, Christoff and Taylor, 1976). This differentiation is associated with decreased clonogenicity in agar (Preisler *et al.*, 1975) and perhaps decreased malignancy as well (Friend *et al.*, 1971). The i.v. inoculation of FLC into mice results in a malignant disease which is characterized by leukaemic cell infiltration of the spleen, bone marrow, liver and lymph nodes (Preisler *et al.*, 1975). The s.c. inoculation of the tissue culture cells results in a tumour which is indistinguishable from a myeloblastoma. Eventually dissemination occurs and the animal dies.

The ability of FLC to differentiate *in vitro* upon exposure to defined chemical agents, together with the ability of the cells to produce a malignant disease when inoculated into mice, suggested that it would be possible to determine whether the observations of induced differentiation *in vitro* had any relevance for the treatment of malignant disease *in vivo*. We here report our initial studies of this proposition. These studies clearly demonstrate that the administration of inducing agents *in vivo* can alter the proliferative characteristics of a malignant disease.

### MATERIALS AND METHODS

*Cells.*—Friend leukaemia cells (line 745A) were cultured as previously described (Preisler and Giladi, 1975). On the 3rd day after passage into fresh media the cells were collected by centrifugation, washed once with phosphate-buffered saline (PBS), resuspended in PBS at $5 \times 10^6$ cells/ml and 0·2 ml inoculated either s.c. or i.v. into 8–12-week-old male DBA2/J mice. Except where indicated there were 10 mice in each experimental group.

*Animals.*—The mice were housed in cages containing 10 animals and allowed food and water *ad lib.* As indicated above, they were inoculated i.p. or s.c. with either PBS or the appropriate inducing agent and weighed twice weekly. Dead animals were weighed and autopsied; the intra-abdominal and intra-thoracic organs were inspected, and then the liver, spleen and kidneys removed and weighed.

*Inducing agents.*—These agents were dissolved in PBS to make the appropriate concentrations as indicated in the text below. The abbreviations used in the text are: N-methyl-acetamide (NMA), dimethylacetamide (DMA), and tetramethylurea (TMU). The inducing agents and the PBS were kept in a refrigerator and glass syringes were used throughout an experiment.

*Clonogenicity assay.*—*In vitro* culture of bone marrow and spleen cell suspensions in plasma clots (Stephenson *et al.*, 1971) was used as a functional test for the presence of leukaemic cells. In our hands neither normal spleens nor normal bone marrow cells form colonies in plasma clots in the absence of added colony-stimulating factor or erythropoietin. Furthermore, we have been unable to demonstrate CSF production by FLC (unpublished). Finally, the morphological appearance of the colonies produced by FLC in plasma clots is distinctly different from those formed by either normal granulocytic or erythroid colony-forming units. Hence we used the number of colonies produced by the bone marrow or spleen cells of mice bearing FLC as an indication of the relative number of leukaemic cells present.

Appropriate dilutions of both spleen and bone marrow cell suspensions were made and used to seed plasma clots (spleen cells at $10^5$/ml final concentration and $2 \times 10^4$/ml of bone marrow cells). The clots were cultured in microtitre wells with 0·1 ml/well. The cultures were incubated at 37°C in a humidified atmosphere consisting of 95% room air and 5% $CO_2$. The number of colonies present was determined 7 days after seeding. A group of 20 or more cells was considered to be a colony. Four to 8 culture wells were counted for each point. Each experiment included a bone marrow and spleen cell suspension from a normal mouse to rule out the possibility of spontaneous colony formation by the residual normal cells present in the leukaemic mice.

*Studies of the kinetics of the effects of NMA on FLC growing* in vivo.—In these two studies mice were inoculated with FLC i.v. and 7 days later (t = 7) 7 mice were sacrificed and the following studies carried out:

1. White blood cell count, differential, haematocrit, reticulocyte count on a blood specimen obtained from each mouse.
2. The spleen, liver, and kidneys of each mouse were inspected and weighed.
3. Bone marrow and spleens were studied cytologically and functionally. Marrow and spleen cell suspensions from each mouse were prepared as previously described (Preisler and Henderson, 1971). The 7 individual spleen cell suspensions and the 7 individual bone marrow cell suspensions were pooled to provide 3 separate spleen cell and 3 separate bone marrow cell suspensions in the following manner: the respective spleen cell suspensions and the marrow cell suspensions of the mice with the 2 largest spleens were combined, as were the spleen and marrow suspensions of the mice with the 2 smallest spleens. The spleen and marrow suspensions of the 3 mice with the spleens of intermediary size were pooled. The cell suspensions were counted and slides were made for differential counting. In experiment 2 the proportion of leukaemic cells present was also assayed by determining the clonogenicity in plasma clots.

On Day 7 (t = 7) additional groups of leukaemic mice were randomly assigned to receive daily i.p. injections of either PBS

(0·01 ml/g body wt.) or NMA (0·01 ml of a 10% solution/g of wt.). In experiment 1, 7 mice received PBS and 14 mice received NMA injections for 1, 2, 3, or 4 days respectively. Seven mice from each group were sacrificed 24 h after their last injection and studied as described above for mice at t = 7. The remaining mice which received 1, 2, 3, or 4 days of NMA therapy were not sacrificed but rather were followed until death, at which time they were autopsied. As a control for these latter studies, 10 mice received a daily inoculation of PBS for 4 days starting on t = 7 and were then followed until death. Experiment 2 was similar to experiment 1 except for the following: (1) the mice received daily NMA injections for 1, 4, 6, or 7 days, and (2) the spleen and bone marrow cell suspensions were cultured in the plasma clot system. In experiment 2 mice began to die on Day 6 (t = 13). Therefore on Days 6 and 7 only the following studies were done: the spleens were weighed (from mice who were sacrificed as well as from those mice which spontaneously died of their disease), and the survival data were determined. The other studies were not done because it was felt that the spontaneous death of diseased mice would spuriously skew the data.

*Differential counting of the cells present in the bone marrow and spleen.*—Slides were made of both marrow and spleen cell suspensions using a cytocentrifuge. The slides were benzidine- and Wright–Giemsa-stained. A single observer in a single-blind fashion determined the proportion of FLC (large basophilic cells), nucleated erythroid cells, myeloblasts and promyelocytes, segmented neutrophils, metamyelocytes and lymphocytes.

*Effects of NMA on normal mice and on murine myeloid leukaemia.*—Three to 4 month old DBA2/J mice were inoculated daily for 7 days with either PBS (0·01 ml/g body wt.) or NMA (0·01 ml of a 10% solution/g body wt.). Mice were sacrificed and the same studies as were described for " kinetics of the effects of NMA " were carried out. Identical studies employing RFMS mice were carried out with the daily injection for 12 days.

Twenty-six RF mice were inoculated with $10^6$ spleen cells from a mouse bearing myeloid leukaemia. Six days later 8 mice were sacrificed and the remaining 18 mice divided into two groups: 9 to receive daily PBS injections and the other 9 to receive daily NMA injection as described above. The experiment was terminated on the day the first mouse died. The animals were studied as described above.

A technical difference from those studies on mice bearing Friend leukaemia was that the number of marrow cells/femur and the differential counts of the spleens and bone marrows were carried out on each mouse rather than on pooled specimens as previously described.

### RESULTS

### *A. Effects of NMA on normal mice*

Normal DBA2/J and RFMS mice were injected with NMA for 7 and 12 days respectively. Table I gives the results. NMA administration resulted in a decrease in splenic weight and an increase in the weights of liver and kidney. The number of cells/femur also declined and there was suggestive evidence for a decline in splenic erythropoiesis, while the proportion of lymphocytes increased. There were no other consistent NMA-induced alterations. All animals appeared to be healthy at the time of sacrifice and the gross morphology of all organs appeared normal.

### *B. Friend leukaemia studies*

*Clinical characteristics of Friend disease.*—Palpable splenomegaly was present in the majority of mice within 10–14 days after i.v. inoculation of FLC. Shortly thereafter the animals appeared ill (hunched up, roughened fur) and many became paraplegic. The mice died several days later. While the median time to death varied somewhat between experiments, within an individual experiment it was usually fairly uniform (Fig. 4).

*Effects of daily drug administration to mice with Friend leukaemia.*—In 10 separate experiments daily, i.p. injection of NMA (0·01 ml/g body wt. of a 10% solution) was begun at either 4 or 7 days after leukaemic cell inoculation and con-

Table I.—*Effect of NMA on Normal Mice*

| | Body wt (g) | Hct. (%) | Spleen wt (mg) | Liver wt (mg) | Spleen cell differential (%)† RBC | Lymphs. | Total cell count $\times 10^{-6}$/ femur |
|---|---|---|---|---|---|---|---|
| DBA2/J—PBS | 25±0·5* | 46±0·4 | 80±7 | 1250±45 | 13±2 | 73±2 | 14·8±0·9 |
| NMA | 25±0·8 | 49±1 | 59±6 | 1378±44 | 11±1 | 83±2 | 12·3±0·8 |
| | | $P<0\cdot025$ | $0\cdot1>P>0\cdot05$ | | | $P<0\cdot005$ | $0\cdot1>P>0\cdot05$ |
| RFMS—PBS | 29±0·8 | 46±1 | 154±12 | 1443±42 | 19±3 | 64±3 | 14·3±0·5 |
| NMA | 31±0·6 | 49±0·8 | 100±4 | 1941±63 | 8±1 | 86±2 | 8·2±0·8 |
| | | $0\cdot1>P>0\cdot05$ | $P<0\cdot01$ | $P<0\cdot01$ | $P<0\cdot02$ | $P<0\cdot01$ | $P<0\cdot01$ |

* Mean ± s.e.

† % of nucleated erythroid precursors and lymphocytes.

Eighteen 4-month-old DBA2/J mice were divided into two groups. Nine mice received PBS (0·01 ml/gm wt) and 9 mice received NMA (0·01 ml of a 10% solution/g) i.p. for 7 days and were then sacrificed. The RFMS were treated the same except that PBS and NMA were administered for 12 days.

tinued until death. Control mice received PBS. In an occasional experiment survival appeared to be prolonged by the administration of NMA. At the time of death, in every experiment the spleens of the NMA-treated mice were always smaller than those of the control mice. Studies employing the same total daily dose of NMA but administered in divided doses (twice a day) yielded identical results (no consistent prolongation of survival but the spleens of treated mice were always significantly smaller than those of the controls; Fig. 1). The twice daily injection of DMA (0·005 ml/g body wt. of a 10% solution) or TMU (0·05 ml/g of a 25% solution) produced the same effects as NMA (Fig. 1).

Since the major drug effect appeared to be on the spleen and since the NMA was being administered by the i.p. route it seemed possible that the effects which we observed might be due to a direct effect on the spleen. To rule this out, experiments which were identical to those described in the preceding paragraph but in which the NMA was injected s.c. were done (6 separate experiments). In each experiment the results were identical to those in which the i.p. route of injection was used (only occasional prolongation of survival, but always inhibitory effects on leukaemic cell proliferation in the spleen).

Fig. 1.—Mice were inoculated i.v. with FLC at t = 0. Beginning on Day 4, the mice were inoculated i.p. with either saline (0·005 ml/g BID), NMA (0·005 ml of 10% solution/g BID), TMU (0·05 ml BID of a 25% solution) or NMA-TMU (0·005 ml NMA in AM and 0·05 ml TMU in PM).

*Kinetics of the effects of NMA*.—Within one day of administration, NMA had demonstrable effects on spleen weight and on the proportion of leukaemic cells in the spleen and bone marrow (Fig. 2*a*). By Day 4 (t = 11) the spleens of the

FIG. 2.—Kinetics of NMA effects. (a) Mean of splenic weights of serially sacrificed mice. (b) Effect of NMA on the proportion of FLC in the spleen. (c) Effect of NMA on the proportion of femoral FLC.

PBS mice were $2\frac{1}{2}$ to $3\frac{1}{2}$ times as large as those of the treated mice. These differences persisted for at least 7 days. It is of interest that during this interval there was virtually no change in the spleen size of the treated mice. Similarly, the proportion of leukaemic cells in the spleens of the treated mice remained quite low during these 4 days while that of the controls progressively increased (Fig. 2*b*). By contrast, the proportion of leukaemic cells in the femoral marrow of both NMA-treated and control mice increased, though the rate of increase in the former was less than in the latter (Fig. 2*c*). The plasma clot clonogenicity studies agree with the histological assessment as to the greater proportion of leukaemic cells in the spleens and femurs of PBS-treated mice. At $t = 7$, the spleen and femurs of the leukaemic mice produced 2 colonies/$10^4$ cells and 14 colonies/$10^3$ cells respectively. After 1 day of PBS or NMA administration the spleens produced 5 and 4 colonies/$10^4$ cells respectively and after 4 days of PBS or NMA administration the number of colonies produced by $10^4$ leukaemic spleen cells was 227 for the PBS- and 63 for the NMA-treated mice. After 4 days of treatment the femurs of PBS-treated mice produced more than 150 colonies/$10^3$ cells (too many colonies to count) while that of the NMA-treated mice produced 65 colonies/$10^3$ cells. There were several other consistent differences between the NMA treated mice and the control mice. The number of bone marrow cells present

(a) (b) (c)

FIG. 3.—Kinetics of NMA effects. (a) Comparison of the number of femoral marrow cells in PBS and NMA treated mice. The % difference was calculated using the following equation:

$$\frac{\text{Average no. femoral cells from NMA treated mice} - \text{average no. femoral cells from PBS treated mice}}{\text{Average no. femoral cells from PBS treated mice}} \times 100$$

(b) Effect of NMA on the proportion of splenic nucleated erythroid cells in mice with Friend leukaemia. (c) Effect of NMA in the proportion of splenic lymphocytes in mice with Friend leukaemia.

in the femurs of the NMA treated mice was greater than that of the controls (Fig. 3*a*). This effect was so apparent that an observer had little difficulty identifying the femur of an NMA-treated mouse on the basis of its gross appearance. With the exception of the proportion of leukaemic marrow cells, there were no other detectable differences between the bone marrows of NMA- and PBS-treated mice. On the other hand, the proportion of erythroid precursors in the spleens of NMA-treated mice declined between Days 1–4 while it increased in the spleens of the control mice (Fig. 3*b*). The proportion of lymphocytes in the NMA-treated mice remained fairly constant while that of the PBS controls declined (Fig. 3*c*). In the PBS-treated mice the data given in Fig. 3*b* and *c* probably represent continued proliferation of FLC resulting in a relative decrease in the number of splenic lymphocytes and an increase in splenic erythroid precursors either as a reaction to the presence of FLC or as the progeny of the FLC themselves. In the NMA-treated mice, inhibition of FLC proliferation prevents the relative decline in lymphocytes and the increase in erythroid precursors. There were no consistent differences in the proportion of either metamyelocytes or mature granulocytes, whilst there was some suggestion of a slight rise in the proportion of myeloblasts and promyelocytes, in the spleens of NMA-treated mice.

There were no consistent differences in the peripheral white blood cell counts, leucocyte differentials, haematocrits, or reticulocyte counts between the NMA-treated mice and the controls.

Figure 4 shows the survival data of the mice in which NMA therapy was administered for 1 or more days and then discontinued and the mice permitted to die of their disease. In both experiments there is a suggestion that the NMA-treated mice survived slightly longer than the PBS controls, particularly for the mice which received NMA for 4 days. Table II gives the splenic weights at expt. 1

expt. 2

Fig. 4.—Effect of NMA on the survival of mice with Friend leukaemia. Mice were treated with NMA for the indicated periods of time and then permitted to die of their disease. Arrow indicates start of treatment.

Table II.—*Effect of a Short Course of NMA Administration on Spleen Wt. at the Time of Death*

| Duration of treatment | Exp. 1 | | Exp. 2 | |
|---|---|---|---|---|
| | PBS | NMA | PBS | NMA |
| 1 | — | 148±49* | — | 317±63 |
| 2 | — | 149±35 | — | — |
| 3 | — | 221±37 | — | — |
| 4 | 224±38 | 219±66† (165±45) | — | 191±43‡ |
| 6 | — | — | — | 168±27 |
| 7 | — | — | 407±32 | 117±16 |

* Mean spleen wt. (mg) ± s.e.

† One mouse in this group survived for 47 days (median for the other 6 mice was 16·5 days). The spleen of this mouse weighed 540 mg. The figures in parenthesis give the mean and standard error if the weight of this mouse's spleen is not included in the calculation.

‡ Average of 6 spleens, one mouse alive at 50 days.

DBA2/J mice bearing Friend leukaemia received daily treatment with either PBS or NMA for the indicated periods of time and then were followed until death (not sacrificed). The survival curves for these mice are given in Fig. 4.

the time of death of the mice in these experiments. Several observations are worthy of consideration. Even though treatment with NMA had been stopped, the splenic size of the NMA-treated mice at the time of death was smaller than that of the control mice. Comparison of the data presented in Table II with that presented in Fig. 2*a* reveals that in the majority of cases there was no significant difference in the spleen sizes between mice which were treated with NMA for a finite period of time and then were sacrificed, and mice which received the same NMA treatment but which were not sacrificed, but were permitted to die as a result of their disease. Since their spleens (the major site of leukaemic proliferation) were smaller, at the time of death the total body tumour load of mice which had received NMA for 1 or more days was less than that of control mice.

*Effect of NMA on the growth of s.c. tumours.*—In a similar experiment, 40 mice were inoculated s.c. with $10^6$ FLC. On Day 4, prior to the appearance of the tumours, the mice were divided into 4 groups of 10 mice. One group received daily i.p. inoculations of PBS while the other three received either NMA, TMU, or NMA/TMU. Figure 5 illustrates the rate of growth of tumours. Eight days after tumour cell inoculation, and 4 days after initiation of drug administration, the tumours in the PBS- and NMA/TMU-treated groups were slightly larger than those of the other 2 groups. Over the next 10 days the rate of increase in tumour size for each of the groups was identical, indicating that neither NMA nor TMU administration inhibited tumour growth. While the survival of the treated mice was less than the controls, the spleen sizes of the TMU- and NMA-treated mice were less than that of the control mice (control 221 ± 61 mg, NMA 31 ± 9 mg, TMU 60 ± 19 mg, and NMA/TMU 124 ± 25 mg) indicating at the time of death a drug effect on leukaemic cells growing in the spleen despite a lack of effect on tumours growing s.c.

Fig. 5.—Effect of NMA, TMU, or NMA + TMU on the growth of FLC s.c. Doses were the same as those used in Fig. 1. The numbers above each indicate the number of surviving mice. The two greatest diameters of each tumour were measured and multiplied to arrive at tumour size. Within each group the size of the tumours were averaged. There were 10 mice in each group. Arrow indicates start of treatment.

After Day 18 the slope of the increase in tumour size of the treated mice became less than that of the controls. This occurred at a time at which the treated mice appeared to be dying of drug toxicity, and may have been secondary to the generally toxic state of the mice.

These observations are similar to other observations made in the course of these studies. In 3 separate studies neither the direct injection of DMSO or DMA (3×/day for 10 days) nor their topical application had an inhibitory effect on the rate of increase in tumour size. In addition, occasional mice who had received i.v. inoculations of FLC, developed lymph node enlargement, tumour growth at the site of FLC inoculation or peri-rectal masses. We have found that tumours growing in these sites continue to enlarge in NMA-treated mice despite the fact that the splenic disease is arrested.

*Other effects of NMA.*—Occasionally the livers of the PBS control mice were mottled in appearance. This was much more frequent in NMA-treated mice and in these latter mice it was not uncommon to detect obvious fatty degeneration.

TABLE III.—*Effect of NMA on Murine Myeloid Leukaemia*

| | Body wt (g) | Hct (%) | Spleen wt (mg) | Liver wt (mg) | Spleen cell differential (%) Myeloblasts | RBC† | Lymphs. | Total cells $\times 10^{-6}$/ femur | % Myeloblasts/ femur |
|---|---|---|---|---|---|---|---|---|---|
| (A) t=0 | 27±0·2* | 44±3 | 421±14 | 1516±58 | 17±2 | 34±1 | 47±2 | 15·6±0·8 | 17±1 |
| (B) t=5d+PBS | 28±1 | 44±1 | 549±46 | 2709±196 | 43±5 | 30±7 | 24±4 | 14·5±2 | 26±4 |
| (C) t=5d+NMA | 29±0·8 | 50±4 | 536±23 | 2613±90 | 36±6 | 38±4 | 22±4 | 14·3±2 | 23±3 |

* Mean ± s.e.

† Nucleated erythroid precursors.

Preliminary pathological studies of these livers are consistent with hepatic necrosis. The NMA-treated mice also lost more weight during the course of their disease than did the control mice (35% vs. 25% of body wt.). In addition, NMA appeared to have some neurological side effects since treated mice often shook when they were picked up. NMA did not alter the gross morphology of the kidneys or kidney wt. NMA appeared to be more toxic to mice bearing Friend leukaemia than to normal mice.

*C. Effects of NMA on murine myeloid leukaemia*

The effects of NMA on murine myeloid leukaemia differed from its effects on Friend leukaemia (Table III). NMA administered for 5 days failed to prevent the progressive splenomegaly which characterizes this disease. NMA also failed to significantly alter the cellular composition of either the bone marrow or spleen, although there was a suggestion of a mild inhibitory effect on myeloblast proliferation.

### DISCUSSION

I.v. inoculation into mice of Friend leukaemia cell line 745A grown in tissue culture results in a malignant disease in which the spleen and bone marrow are the major sites of leukaemic cell proliferation. Occasionally, and most frequently in the mice which survive the longest, there is leukaemic cell proliferation in lymph nodes, and in rare instances there are subcutaneous tumours at the site of leukaemic cell inoculation.

The administration of NMA, DMA, or TMU results in major alterations in the proliferative characteristics of the disease. In these treated mice the splenic disease was arrested and there also appeared to be a retardation in the proliferation of the leukaemic cells in the bone marrow. The significance of this latter effect is unclear since it appears that the total number of marrow cells was greater in the treated mice and if one multiplies the percentage of leukaemic marrow cells by the total number 'of marrow cells the difference between the NMA- and PBS-treated mice diminishes. These effects appear to be similar to the effects of NMA on normal haematopoietic elements. When administered to normal mice, NMA produced a decrease both in splenic wt. and in the number of femoral marrow cells. It would appear that the apparent increase in splenic lymphocytes is relative rather than absolute and results from a decrease in the other haemotopoietic elements.

The mechanism of action of these compounds is unclear since we found no evidence of induction of differentiation of the Friend leukaemia cells *in vivo*. Rather, the effects appeared to be due to an inhibition of Friend leukaemia cell proliferation—perhaps similar to the effects of NMA on the haematopoietic elements of normal mice. These effects could be the result of a direct drug effect on the proliferating cells themselves (possibly due to a decrease in intracellular nucleotide pools (Preisler and Rustum, 1975)) or, alternatively, the drug could influence the microenvironment in which the cells are proliferating. Changes in the microenvironment could then affect cell proliferation. Microenvironmental influence on leukaemic cell proliferation have been previously reported (Metcalf and Moore, 1970). In favour of this hypothesis is the lack of effect on NMA on Friend leukaemia cells proliferating s.c.

Since NMA affected normal DBA2/J mice and RFMS mice in an identical fashion, the difference between the effects of NMA on Friend leukaemia cells and on murine myeloid leukaemia must be a result of the differences between the leukaemia cells, and not due to differences in the effects of NMA on these two strains of mice. In favour of this possibility is the lack of NMA effect on Friend leukaemia cells proliferating s.c. since the tumours produced at this site are devoid of erythroid elements and are indistinguishable from myeloblastomata (Preisler *et al.*, 1975) whereas Friend leukaemia cells proliferating in the spleen produce erythroid elements, as previously described.

The observations that NMA administration resulted in an increase in the haematocrits of normal mice and mice bearing myeloid leukaemia deserves further comment. This apparent increase cannot be ascribed to dehydration since there was no difference in the weights of the animals. Furthermore in preliminary studies we have observed that the administration of DMSO to post-hypobaric polycythaemic mice resulted in an increase in $^{59}$Fe incorporation into red cells, an effect which appeared to be blocked by anti-erythropoietin antibody (unpublished). These effects were not observed in mice bearing Friend leukaemia, perhaps because the disease itself appears to adversely affect normal erythropoiesis and result in anaemia. Nevertheless, the apparent increase in circulating red cell mass appears to be a paradox in view of the apparent NMA-induced decrease in haematopoietic elements in both spleen and bone marrow and in the absence of a reticulocytosis. While these observations, along with those of inhibition of Friend leukaemia with little effect on myeloid leukaemia suggest a relationship between NMA effect and erythropoiesis, the nature of this relationship awaits further definition.

Despite altering the nature of the disease and producing a marked reduction in leukaemic cell body burden, these agents did not consistently prolong survival of leukaemic mice. Two possible explanations suggest themselves. Perhaps there was no prolongation of survival because the tumour cells were proliferating in a site " protected " from the effects of NMA. For example, proliferation of leukaemic cells in the spinal cord may have been unaffected by NMA because of " microenvironmental " resistance. The mouse would then become paraplegic and die of thirst and starvation.

An alternate explanation relates to the hepatic toxicity of NMA. The livers of treated mice (bearing Friend leukaemia) were frequently enlarged and yellow. Preliminary pathology studies are consistent with hepatic necrosis. It is possible that the beneficial affects of the reduction in tumour load were counterbalanced by the hepatotoxic effects of NMA. Perhaps the apparent beneficial effects of 4 days of therapy reflects a fortuitous balance between the hepatotoxic and anti-Friend leukaemia effects of NMA.

In any event, agents which induce the differentiation of Friend leukaemia cells *in vitro* can also alter the proliferation of these cells *in vivo*. While the mechanism of action and the optimal mode of administration are not known, these agents may represent a new class of antileukaemic compounds.

This research was supported by USPHS Grants CA-5834 and CA-17785.

We are grateful to Gregory Christoff, Bradley Bryant, Shakerun Alarmru, and Steven Gottlieb for their technical assistance. We would also like to thank Dr John Storer of Oak Ridge National Laboratories for assisting us in establishing a colony of leukaemic RFMS mice at Roswell Park Memorial Institute.

### REFERENCES

DeCosse, J. J., Gossens, C. L., Kuzma, J. F. & Unsworth, B. R. (1973) Breast Cancer: Induction of Differentiation by Embryonic Tissue. *Science, N.Y.*, **181**, 1057.

Editorial, Neuroblastoma (1975) *Lancet*, i, 379.

Friend, C., Scher, W., Holland, J. G. & Sato, T. (1971) Hemoglobin Synthesis in Murine Virus-induced Leukemic Cells *In Vitro*: Stimulation of Differentiation by Dimethylsulfoxide. *Proc. natn. Acad. Sci. U.S.A.*, **68**, 378.

Goldstein, N. M., Burdman, J. A. & Journey, L. J. (1964) Long-term Tissue Culture of Neuroblastomas. II. Morphological Evidence of Differentiation and Maturation. *J. natn. Cancer Inst.*, **32**, 165.

Ichikawa, Y. (1969) Differentiation of a Cell Line of Myeloid Leukemia. *J. cell. Physiol.*, **74**, 223.

LEHMAN, J. M., SPEERS, W. C., SWARTZENDRUBER, D. E. & PIERCE, G. B. (1974) Neoplastic Differentiation: Characteristics of Cell Lines Derived from a Murine Teratocarcinoma. *J. cell. Physiol.*, **84**, 13.

METCALF, D. & MOORE, M. A. S. (1970) Factors Modifying Stem Cell Proliferation of Myelomonocytic Leukemia Cells *In Vitro* and *In Vivo*. *J. natn. Cancer Inst.*, **44**, 801.

PIERCE, G. B. & WALLACE, C. (1971) Differentiation of Malignant to Benign Cells. *Cancer Res.*, **31**, 127.

PRASAD, K. N. (1972*a*) Morphologic Differentiation Induced by Prostaglandin in Mouse Neuroblastoma Cells in Culture. *Nature, New Biol.*, **236**, 49.

PRASAD, K. N. (1972*b*) Cyclic AMP-induced Differentiated Mouse Neuroblastoma Cells Lose Tumourigenic Characteristics. *Cytobios.*, **6**, 163.

PRASAD, K. N., GILMER, K. & KUMAR, S. (1973) Morphologically " Differentiated " Mouse Neuroblastoma Cells Induced by Noncyclic AMP Agents: Levels of Cyclic AMP, Nucleic Acid, and Protein. *Proc. Soc. expl. Biol. Med.*, **143**, 1168.

PREISLER, H. D., BJORNSSON, S., MORI, M. & BARCOS, M. (1976) Granulocyte Differentiation by Friend Leukemia Cells. *Cell Differentiation* **4**, 273,

PREISLER, H. D., CHRISTOFF, G. & TAYLOR, E. (1976) Cryoprotective Agents as Inducers of Erythroleukemic Cell Differentiation *In Vitro*. *Blood*, **47**, 363.

PREISLER, H. D. & GILADI, M. (1975) Differentiation of Erythroleukemic Cells *In Vitro:* Irreversible Induction by Dimethyl Sulfoxide. *J. cell. Physiol.*, **85**, 537.

PREISLER, H. D. & HENDERSON, E. S. (1971) The Effect of 1,3-Bis (2 chloroethyl)-1-nitrosurea (BCNU) and Cytosine Arabinoside (ara-C) on Hematopoiesis in the Mouse. *J. natn. Cancer Inst.*, **47**, 971.

PREISLER, H. D., LUTTON, J. D., GILADI, M., GOLDSTEIN, K. & ZANJANI, E. D. (1975) Loss of Clonogenicity in Agar by Differentiating Erythroleukemic Cells. *Life Sciences*, **16**, 1241.

PREISLER, H. D. & LYMAN, G. (1975) Differentiation of Erythroleukemia Cells *In Vitro:* Properties of Chemical Inducers. *Cell Differ.*, **4**, 179.

PREISLER, H. D. & RUSTUM, Y. (1975) Differing Effects of Inducers of Differentiation on the Ribonucleotide Pool Sizes of Friend Leukemia Cells. *Life Sciences*, **17**, 1287.

SILAGI, S. & BRUCE, S. A. (1970) Suppression of Malignancy and Differentiation in Melanotic Melanoma Cells. *Proc. natn. Acad. Sci., U.S.A.*, **66**, 72.

SINKS, L. (1973) In *Cancer Medicine* Ed. J. F. Holland and E. Frei. Philadelphia: Lea & Febiger. p. 1893.

STEPHENSON, J. R., AXELRAD, A. A., MCLEOD, D. L. & SHREEVE, M. M. (1971) Induction of Colonies of Hemoglobin Synthesizing Cells by Erythropoietin *In Vitro*. *Proc. natn. Acad. Sci. U.S.A.*, **68**, 1542.